# A machine learning approach to distinguish between knees without and with osteoarthritis using MRI-based radiomic features from tibial bone

**DOI:** 10.1007/s00330-021-07951-5

**Published:** 2021-04-21

**Authors:** Jukka Hirvasniemi, Stefan Klein, Sita Bierma-Zeinstra, Meike W. Vernooij, Dieuwke Schiphof, Edwin H. G. Oei

**Affiliations:** 1 Department of Radiology & Nuclear Medicine, Erasmus MC University Medical Center, P.O. Box 2040, CA 3000 Rotterdam, The Netherlands; 2 Department of General Practice, Erasmus MC University Medical Center, Rotterdam, The Netherlands; 3 Department of Orthopedics, Erasmus MC University Medical Center, Rotterdam, The Netherlands; 4 Department of Epidemiology, Erasmus MC University Medical Center, Rotterdam, The Netherlands

**Keywords:** Magnetic resonance imaging, Radiomics, Knee osteoarthritis, Bone, Machine learning

## Abstract

**Objectives:**

Our aim was to assess the ability of semi-automatically extracted magnetic resonance imaging (MRI)–based radiomic features from tibial subchondral bone to distinguish between knees without and with osteoarthritis.

**Methods:**

The right knees of 665 females from the population-based Rotterdam Study scanned with 1.5T MRI were analyzed. A fast imaging employing steady-state acquisition sequence was used for the quantitative bone analyses. Tibial bone was segmented using a method that combines multi-atlas and appearance models. Radiomic features related to the shape and texture were calculated from six volumes of interests (VOIs) in the proximal tibia. Machine learning–based Elastic Net models with 10-fold cross-validation were used to distinguish between knees without and with MRI Osteoarthritis Knee Score (MOAKS)–based tibiofemoral osteoarthritis. Performance of the covariate (age and body mass index), image features, and combined covariate + image features models were assessed using the area under the receiver operating characteristic curve (ROC AUC).

**Results:**

Of 665 analyzed knees, 76 (11.4%) had osteoarthritis. An ROC AUC of 0.68 (95% confidence interval (CI): 0.60–0.75) was obtained using the covariate model. The image features model yielded an ROC AUC of 0.80 (CI: 0.73–0.87). The model that combined image features from all VOIs and covariates yielded an ROC AUC of 0.80 (CI: 0.73–0.87).

**Conclusion:**

Our results suggest that radiomic features are useful imaging biomarkers of subchondral bone for the diagnosis of osteoarthritis. An advantage of assessing bone on MRI instead of on radiographs is that other tissues can be assessed simultaneously.

**Key Points:**

*• Subchondral bone plays a role in the osteoarthritis disease processes.*

*• MRI radiomics is a potential method for quantifying changes in subchondral bone.*

*• Semi-automatically extracted radiomic features of tibia differ between subjects without and with osteoarthritis.*

**Supplementary Information:**

The online version contains supplementary material available at 10.1007/s00330-021-07951-5.

## Introduction

Osteoarthritis is the most common joint disease affecting over 40 million people in Europe [1]. It reduces the quality of life of an individual and imposes a large economic burden on the society, since the direct and indirect costs can be as high as 2.5% of the gross domestic product of a nation [2, 3]. Osteoarthritis affects all tissues in the joint, e.g., causing progressive degeneration of articular cartilage and changes in the subchondral bone density and structure [4, 5]. Macroscopic alterations in the subchondral bone include osteophytes, bone sclerosis (stiffening), and cysts [4, 5]. Advances in osteoarthritis diagnostics, prevention, and treatment will have a major impact on patients and society.

It has been recognized that subchondral bone plays a role in the pathogenesis of osteoarthritis [5–7] and subchondral bone has been suggested as a target for potential disease-modifying osteoarthritis drugs [8]. However, the exact role of subchondral bone in the development and progression of osteoarthritis is still unclear [5].

Magnetic resonance imaging (MRI) is considered the most comprehensive imaging modality for knee osteoarthritis assessment [9]. Semi-quantitative scoring systems have been developed to assess osteoarthritis-related structural deterioration of tissues on MRI [10]. Furthermore, many quantitative methods to assess articular cartilage and meniscus exist [11–13]. As subchondral bone is also involved in osteoarthritis disease processes, quantitative imaging biomarkers from bone might be helpful in the detection, prediction, and monitoring of the disease.

Radiomics is a field where a large number of quantitative image features (features related to intensity, geometric shape, and texture) are extracted from an image and correlated to biological markers and clinical outcomes. Radiomic methods have been successfully applied to different MRI data [14] but these methods have not yet been widely used for the assessment of knee osteoarthritis. Bone shape and texture variables have been previously extracted from knee MRI to assess osteoarthritic changes [15–18] and texture has been shown to correlate with the actual three-dimensional microstructure of subchondral bone [19]. However, previous texture analysis studies have manually defined regions of interests, analyzed limited number of slices, and used a limited number of texture variables.

As subchondral bone plays a role in the osteoarthritis disease processes, the aim of this study was to semi-automatically extract radiomic features from tibial subchondral bone using knee MRI data and to assess the ability of these radiomic features to distinguish between knees without and with osteoarthritis in a large population-based cohort.

## Subjects and methods

### Study cohort

The study data consisted of baseline data of 665 female participants from a sub-study (RS-III-1) of the Rotterdam Study, a population-based study in the Netherlands that investigates prevalence, incidence, and risk factors for various chronic disabling diseases [20–22]. Of the participants of the RS-III-1, the first 1116 females aged 45–60 years were invited to join a sub-study investigating early signs of knee osteoarthritis [20, 21]. Of the 891 females who agreed to participate, 665 females with sufficient MR image quality and visual MRI grades available were included in the current study. The mean age and body mass index (BMI) of the subjects were 54.6 (standard deviation (SD): 3.7) years and 26.8 (SD: 4.6) kg/m^2^, respectively. The Medical Ethics Committee of Erasmus University Medical Center approved the study and all subjects provided written informed consent.

### MRI acquisition

All participants were scanned with a 1.5-T MRI scanner (Signa Excite 2, General Electric Healthcare) using an eight-channel cardiac coil that allowed imaging of both knees at once without repositioning the subject. The scanning protocol consisted of sagittal dual-echo fast spin echo (FSE) proton density–weighted, FSE T2-weighted with fat suppression, spoiled gradient echo with fat suppression, and fast imaging employing steady-state acquisition (FIESTA) sequences (Table 1).

**Table 1:** Parameters of the magnetic resonance imaging protocol

Sequence	TR (ms)	TE (ms)	FOV (mm)	Matrix	Flip angle (degrees)	Slice thickness/spacing (mm)
Dual echo FSE PD	2700	16	160*160	512*512	90	3.2/3.2
FSE T2-weighted FS	4100	59	160*160	512*512	90	3.2/3.2
SGE FS	27	6.3	160*160	512*512	30	3.2/1.6
FIESTA	5.6	1.8	160*160	512*512	35	1.2/0.6

*FIESTA*, fast imaging employing steady-state acquisition; *FS*, fat suppression; *FSE*, fast spin echo; *FOV*, field of view; *PD*, proton density; *SGE*, spoiled gradient echo; *TE*, time to echo; *TR*, repetition time

### MRI assessment

Two experienced readers scored the MRIs using the MRI Osteoarthritis Knee Score (MOAKS) [10]. The readers were extensively trained by an experienced musculoskeletal radiologist (E.O., > 15 years of experience with musculoskeletal MRI in clinical and research settings), as described previously [23].

We used a previously proposed definition for the identification of tibiofemoral osteoarthritis on MRI [24, 25]. Tibiofemoral osteoarthritis was defined as the presence of a definite osteophyte and full-thickness cartilage loss, or one of these features and two of the following features: (1) subchondral bone marrow lesion or cyst not associated with meniscal or ligamentous attachments, (2) meniscal maceration or degeneration (including a horizontal tear), or (3) partial thickness cartilage loss [24]. Grade 1 and 2 cartilage lesions were classified as partial thickness lesions and grade 3 lesions as full-thickness lesions. Grade 2 and 3 osteophytes were classified as definite osteophytes. Bone marrow lesions and cysts were present when scored as grade 1 or higher. Meniscus-associated features were present when maceration or degeneration had grade 1 or higher or a horizontal tear was present. We did not assess meniscal subluxation or bone attrition.

In addition to the tibiofemoral osteoarthritis, the ability of radiomic features to distinguish between knees without and with medial tibial cartilage damage, osteophytes, and bone marrow lesions were analyzed. A subject was included in the medial tibial cartilage damage group if she had any cartilage loss (grade 1 or higher for the size of any cartilage loss) in the medial anterior, central, or posterior tibia. Similarly, a subject was included in the medial tibial osteophytes group if she had osteophytes in the medial tibia (grade 1 or higher) and in the bone marrow lesion group if she had any bone marrow lesions (grade 1 or higher for the size of bone marrow lesions) in the medial anterior, central, or posterior part of tibia.

### Automatic segmentation of tibia

The FIESTA scans were used in the quantitative analyses. Tibial bones from the right knees of the participants were segmented using an in-house automatic segmentation method that combines multi-atlas and appearance models [26, 27]. Twenty manually segmented tibias were used as atlases for the multi-atlas model and as training data for the appearance model. In the multi-atlas part, the atlas images were registered to the target image (i.e., the image to be segmented) using the Elastix software [28]. The registration was done by estimating an affine transformation followed by a non-rigid transformation. Mutual information was used as similarity measure for both transformations. The obtained registration parameters were used to deform the manual segmentations of the atlas images. The probability of a voxel in the target image to be part of the tibia segmentation or the background (i.e., non-tibia) was computed by averaging the deformed segmentations of all atlas images. The appearance model consisted of a random forest classifier that was trained on Gaussian scale space features in the training set. The feature vector (49 features) consisted of the intensity values and Gaussian-filtered version of the original images, first-order and second-order Gaussian derivatives in every axis direction, the gradient magnitude, the Laplacian, Gaussian Curvature, and the three eigenvalues of the Hessian of the Gaussian-filtered images. The classifier was then used to classify voxels on the target image to be either tibia or background. The final output of the segmentation method was obtained by combining the probabilities of the multi-atlas and appearance components. The segmentations were visually inspected and manually corrected if needed.

**Fig. 1:**
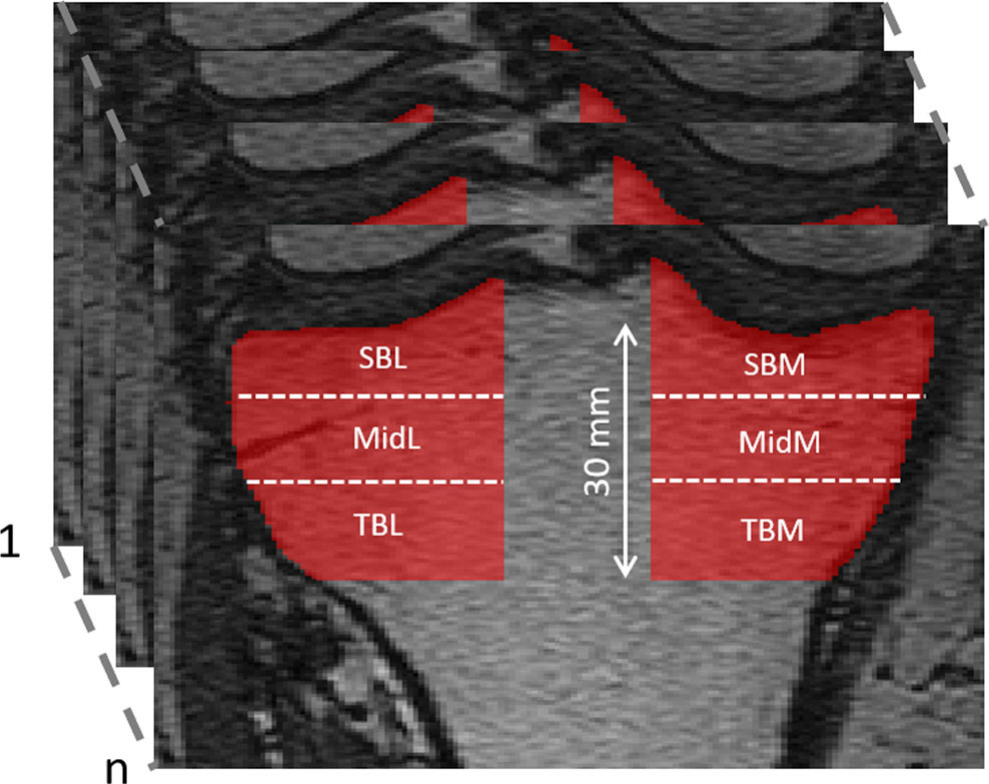
3-D volumes of interest (VOIs) were extracted from medial (SBM) and lateral subchondral bone (SBL), mid-part of medial (MidM) and lateral (MidL) compartments, and medial (TBM) and lateral trabecular bone (TBL)

Altogether six three-dimensional volumes of interest (VOI) were automatically extracted from the medial and lateral compartments of the tibia (Fig. 1). Medial and lateral tibial spines were located by searching the highest and the second-highest coordinates along the vertical axis of the segmented sagittal slices. Because the positioning of the knee was similar among all scans, the medial and lateral compartments were identified using the tibial spines and outer borders of the tibia as landmarks. Medial and lateral subchondral bone VOIs were placed immediately below the cartilage-bone interface. The bottom coordinates for the abovementioned VOIs were defined as 10 mm below the cartilage-bone interface on the middle point of each compartment. Medial and lateral mid-part VOIs were placed under the subchondral bone VOIs. Medial and lateral trabecular bone VOIs were placed under the middle region VOIs. The height of the mid-part and trabecular bone VOIs was 10 mm. It should be noted that despite referring the VOIs to as the subchondral bone, mid-part, and trabecular bone VOIs, different bone types are mixed in the VOIs [29, 30].

### Radiomics

Radiomic features that are related to the shape and texture of the region were calculated from each VOI using the open-source Workflow for Optimal Radiomics Classification package (v. 3.0.0) in Python [31]. Seventeen shape features, 3 orientation features, 12 histogram-based features, and 271 texture features were extracted (Supplementary Table 1). Texture features included local binary patterns, gray level co-occurrence matrix, gray level run length matrix, gray level size zone matrix (GLSZM), neighborhood gray tone difference matrix, and Gabor features. Features associated with shape were calculated only for the whole tibial volume.

### Statistical analyses

To assess the accuracy of the automatic segmentation, the Dice similarity coefficient [32] was used to determine the overlap between five automatically and manually segmented tibias. These tibias were not included in the atlas set. The Dice coefficient ranges between 0 and 1 and higher value indicates better segmentation accuracy.

Machine learning was used for dimensionality reduction and to evaluate how well subjects without and with osteoarthritis can be distinguished using radiomics features from tibial bone. Three different models were used: (1) covariate model that included age and BMI that are two common risk factors for osteoarthritis [33], (2) image features model, and (3) combined covariate + image features model. Dimensionality reduction and classification were done using the Elastic Net regression, which is a regularized logistic regression method [34, 35]. The Elastic Net linearly combines the L1 and L2 penalties of lasso and ridge regression methods. To optimize the ratio of the L1 and L2 penalties (*α*) and the strength of the penalty parameter (*λ*) of the Elastic Net, 10-fold cross-validation with a grid search and 100 repetitions was performed. In the grid, the values of *α* varied from 0.1 to 1 with an increment of 0.05 and *λ* from 0.001 to 0.15 with an increment of 0.009. When *α* is close to 0, the Elastic Net approaches ridge regression, while when *α* is 1, lasso regression is performed. In cross-validation, the performance of the aforementioned three models to distinguish between subjects without and with osteoarthritis was assessed using the area under the receiver operating characteristic (ROC AUC) and precision-recall curves (PR AUC) along with 95% confidence intervals (CI) [36]. To assess the ability of radiomic features from medial tibial bone to distinguish individual osteoarthritis-related structural changes, analyses were repeated using medial tibial cartilage damage, osteophytes, and bone marrow lesions as an outcome variable. The Elastic Net experiments were done using the R software (version 3.5.2) with Caret [37], PRROC [38], glmnet [34], and precrec [39] packages.

## Results

Of the 665 analyzed knees, 76 (11.4%) had tibiofemoral osteoarthritis, 91 (13.7%) had medial tibial cartilage damage, 85 (12.8%) had a medial tibial osteophyte, and 70 (10.5%) had a medial tibial bone marrow lesion. The mean Dice similarity coefficient for the uncorrected automatic segmentation of the tibia was 0.96 (SD: 0.02). Of the automatically segmented knees, 86 (12.9%) were manually corrected.

**Fig. 2:**
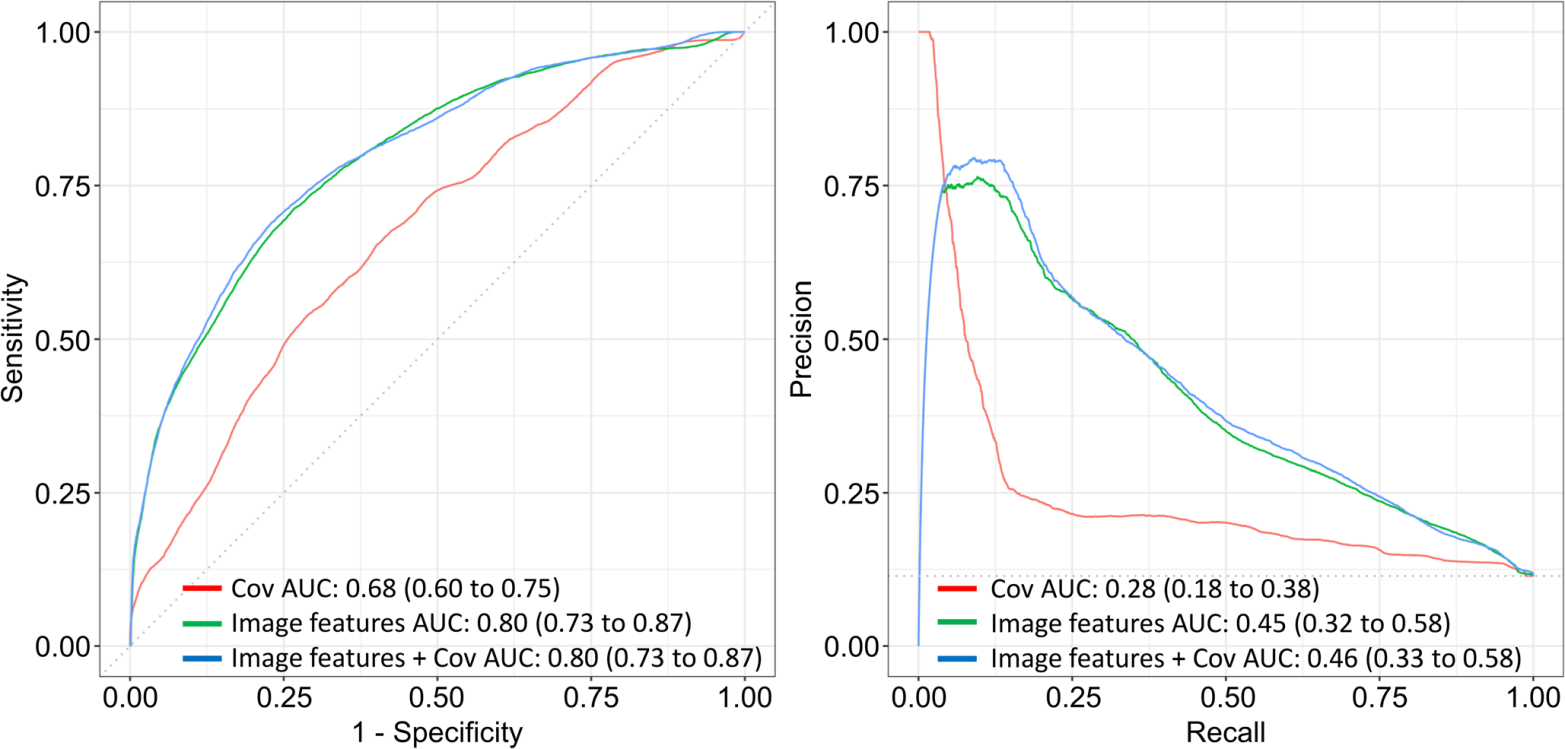
Receiver operating characteristic and precision-recall curves and respective area under the curve (AUC) values to distinguish between knees without and with osteoarthritis using models with covariates (age and body mass index), image features from all VOIs, and covariates and image features in the same model

When classifying knees without and with tibiofemoral osteoarthritis, ROC AUC and PR AUC of 0.80 (95% CI: 0.73–0.87) and 0.46 (95% CI: 0.33–0.58) were obtained with the model that combined image features from all VOIs and covariates (Table 2, Fig. 2). When each VOI was assessed separately, the medial subchondral bone VOI had the highest ROC AUC (Table 2). The model that combined image features from the medial subchondral bone VOI with covariates had an ROC AUC of 0.80 (95% CI: 0.72–0.87) and a PR AUC of 0.46 (95% CI: 0.33–0.58) (Supplementary Figure 1).

**Table 2:** Area under the receiver operating characteristic curve (ROC AUC) and area under the precision-recall curve (PR AUC) values to distinguish between knees without and with osteoarthritis using models with only covariates (age and body mass index), only image features, and combined covariates and image features model for different volumes of interests (VOIs)

VOI	Covariates		Image features		Covariates + image features	
	ROC AUC	PR AUC	ROC AUC	PR AUC	ROC AUC	PR AUC
All	0.68 (0.60–0.75)	0.28 (0.18–0.38)	0.80 (0.73–0.87)	0.45 (0.32–0.58)	0.80 (0.73–0.87)	0.46 (0.33–0.58)
SBM	0.68 (0.60–0.75)	0.28 (0.18–0.38)	0.79 (0.71–0.86)	0.46 (0.33–0.58)	0.80 (0.72–0.87)	0.46 (0.33–0.58)
MidM	0.68 (0.60–0.75)	0.28 (0.18–0.38)	0.77 (0.70–0.84)	0.39 (0.28–0.51)	0.78 (0.71–0.86)	0.41 (0.33–0.58)
TBM	0.68 (0.60–0.75)	0.28 (0.18–0.38)	0.74 (0.67–0.81)	0.33 (0.22–0.44)	0.76 (0.68–0.83)	0.35 (0.24–0.46)
SBL	0.68 (0.60–0.75)	0.28 (0.18–0.38)	0.72 (0.65–0.80)	0.32 (0.21–0.43)	0.74 (0.67–0.81)	0.35 (0.23–0.46)
MidL	0.68 (0.60–0.75)	0.28 (0.18–0.38)	0.73 (0.66–0.80)	0.33 (0.22–0.44)	0.75 (0.68–0.82)	0.35 (0.24–0.47)
TBL	0.68 (0.60–0.75)	0.28 (0.18–0.38)	0.74 (0.66–0.81)	0.34 (0.23–0.45)	0.76 (0.69–0.83)	0.37 (0.25–0.49)

*All*, all VOIs in the same model; *SBM*, medial subchondral bone VOI; *MidM*, medial mid-part VOI; *TBM*, medial trabecular bone VOI; *SBL*, lateral subchondral bone; *MidL*, lateral mid-part VOI; *TBL*, lateral trabecular bone VOI

When classifying knees without and with medial tibial cartilage damage, the model that included image features from the medial subchondral bone VOI and covariates had the highest ROC AUC and PR AUC (0.66 (95% CI: 0.59–0.73) and 0.30 (95% CI: 0.21–0.39)) (Table 3, Supplementary Figure 2).

**Table 3:** Area under the receiver operating characteristic curve (ROC AUC) and area under the precision-recall curve (PR AUC) values to distinguish between knees without and with medial tibial cartilage damage with the models using only covariates (age and body mass index), image features, and combined covariates and image features model for different volumes of interests (VOIs)

VOI	Covariates		Image features		Covariates + image features	
	ROC AUC	PR AUC	ROC AUC	PR AUC	ROC AUC	PR AUC
All medial	0.60 (0.52–0.69)	0.21 (0.15–0.27)	0.62 (0.54–0.69)	0.27 (0.18–0.36)	0.62 (0.54–0.70)	0.27 (0.18–0.36)
SBM	0.60 (0.52–0.69)	0.21 (0.15–0.27)	0.66 (0.59–0.73)	0.29 (0.20–0.38)	0.66 (0.59–0.73)	0.30 (0.21–0.39)
MidM	0.60 (0.52–0.69)	0.21 (0.15–0.27)	0.58 (0.50–0.66)	0.22 (0.15–0.29)	0.59 (0.51–0.67)	0.22 (0.15–0.30)
TBM	0.60 (0.52–0.69)	0.21 (0.15–0.27)	0.55 (0.47–0.63)	0.20 (0.13–0.26)	0.55 (0.48–0.63)	0.20 (0.14–0.26)

*All medial*, all medial VOIs in the same model; *SBM*, medial subchondral bone VOI; *MidM*, medial mid-part VOI; *TBM*, medial trabecular bone VOI

When classifying knees without and with medial tibial osteophytes, an ROC AUC of 0.80 (95% CI: 0.73–0.86) and a PR AUC of 0.45 (95% CI: 0.34–0.57) were obtained with the model that included image features from all medial side VOIs and covariates (Table 4, Supplementary Figure 3). When each VOI was assessed separately, medial mid-part VOI had the highest ROC AUC and PR AUC (0.79 (95% CI: 0.73–0.89) and 0.44 (95% CI: 0.33–0.56)) (Table 4).

**Table 4:** Area under the receiver operating characteristic curve (ROC AUC) and area under the precision-recall curve (PR AUC) values to distinguish between knees without and with medial tibial osteophytes with the models using only covariates (age and body mass index), image features, and combined covariates and image features model for different volumes of interests (VOIs)

VOI	Covariates		Image features		Covariates + image features	
	ROC AUC	PR AUC	ROC AUC	PR AUC	ROC AUC	PR AUC
All medial	0.64 (0.56–0.72)	0.28 (0.18–0.37)	0.80 (0.73–0.86)	0.45 (0.34–0.57)	0.80 (0.73–0.86)	0.45 (0.34–0.57)
SBM	0.64 (0.56–0.72)	0.28 (0.18–0.37)	0.76 (0.69–0.83)	0.41 (0.29–0.53)	0.77 (0.69–0.84)	0.43 (0.31–0.55)
MidM	0.64 (0.56–0.72)	0.28 (0.18–0.37)	0.79 (0.73–0.86)	0.45 (0.33–0.56)	0.79 (0.73–0.89)	0.44 (0.33–0.56)
TBM	0.64 (0.56–0.72)	0.28 (0.18–0.37)	0.73 (0.66–0.80)	0.34 (0.24–0.44)	0.73 (0.65–0.80)	0.37 (0.26–0.48)

*All medial*, all medial VOIs in the same model; *SBM*, medial subchondral bone VOI; *MidM*, medial mid-part VOI; *TBM*, medial trabecular bone VOI

When classifying knees without and with medial tibial bone marrow lesions, the model that included image features from the medial mid-part VOI and covariates had the highest ROC AUC and PR AUC (0.69 (95% CI: 0.60–0.77) and 0.28 (95% CI: 0.17–0.38)) (Table 5, Supplementary Figure 4).

**Table 5:** Area under the receiver operating characteristic curve (ROC AUC) and area under the precision-recall curve (PR AUC) values to distinguish between knees without and with medial tibial bone marrow lesions with the models using only covariates (age and body mass index), image features, and combined covariates and image features model for different volumes of interests (VOIs)

VOI	Covariates		Image features		Covariates + image features	
	ROC AUC	PR AUC	ROC AUC	PR AUC	ROC AUC	PR AUC
All medial	0.63 (0.54–0.72)	0.24 (0.14–0.34)	0.68 (0.59–0.76)	0.23 (0.15–0.32)	0.68 (0.60–0.77)	0.25 (0.16–0.35)
SBM	0.63 (0.54–0.72)	0.24 (0.14–0.34)	0.65 (0.57–0.73)	0.21 (0.13–0.29)	0.67 (0.59–0.75)	0.25 (0.15–0.34)
MidM	0.63 (0.54–0.72)	0.24 (0.14–0.34)	0.67 (0.59–0.76)	0.25 (0.15–0.34)	0.69 (0.60–0.77)	0.28 (0.17–0.38)
TBM	0.63 (0.54–0.72)	0.24 (0.14–0.34)	0.57 (0.48–0.66)	0.19 (0.11–0.27)	0.58 (0.49–0.68)	0.21 (0.12–0.30)

*All medial*, all medial VOIs in the same model; *SBM*, medial subchondral bone VOI; *MidM*, medial mid-part VOI; *TBM*, medial trabecular bone VOI

The values for *α* and *λ* hyperparameters and five variables with largest coefficients in the best performing Elastic Net models for classifying knees without and with tibiofemoral osteoarthritis, medial tibial cartilage damage, medial tibial osteophytes, and medial tibial bone marrow lesions are presented in Supplementary Table 2.

## Discussion

In this study, we applied a semi-automatic segmentation method and extracted radiomic features from tibial bone using knee MRI data of a large population-based cohort. The highest ROC AUC and PR AUC for classifying knees without and with tibiofemoral osteoarthritis were obtained when all VOIs were combined in the same model. When each VOI was used separately in the model, image features from the medial subchondral bone and mid-part VOIs had the highest ROC AUC and PR AUC. This can be explained by the fact that the medial side of the knee is more commonly affected in osteoarthritis than the lateral compartment [40, 41].

For comprehensiveness, we also tested if the radiomic features are able to distinguish between subjects without and with medial tibial cartilage damage, osteophytes, and bone marrow lesions. Image features from the medial subchondral bone VOI had the highest ROC AUC and PR AUC to distinguish between knees without and with medial tibial cartilage damage indicating an interplay between cartilage and subchondral bone. Image features from the medial mid-part VOI had the highest ROC AUC and PR AUC to distinguish between knees without and with medial tibial bone marrow lesions. The model that combined all medial side VOIs in the same model had the highest ROC AUC and PR AUC to distinguish between knees without and with medial tibial osteophytes. These findings suggest that in addition to the subchondral bone area closest to the cartilage-bone interface, the characteristics of deeper bone areas are also altered in osteoarthritis.

The number of studies using radiomic approach for assessment of bone and osteoarthritis is limited, but texture variables have been previously extracted from knee MRI to assess osteoarthritic changes in bone [16, 17, 19]. One study predicted progression of knee osteoarthritis over 36 months (defined using joint space narrowing on plain radiographs) using bone texture on MRI and reported ROC AUCs between 0.58 and 0.68 depending on the model [15]. Radiography-based bone texture has been used to predict progression or development of osteoarthritis (ROC AUCs between 0.65 and 0.85) [42–44] and to distinguish between knees without and with osteoarthritis (ROC AUCs between 0.81 and 0.85) [45, 46]. The advantage of assessing bone on MRI is that other tissues involved in osteoarthritis can be assessed simultaneously. Furthermore, a two-dimensional plain radiograph is a projection of a three-dimensional structure, whereas MRI can produce three-dimensional data from the target structure.

When looking at the variables related to the geometrical shape of the bone, the compactness variable was included in all best performing models for classifying each of the used outcomes. The compactness variable describes how compact the bone shape on the sagittal slice is compared to a circle [14, 47]. By inspecting the direction of the coefficients in the final Elastic Net regression models, our results indicate that lower compactness is associated with tibiofemoral osteoarthritis.

Although many texture features contributed to the final models, Gabor and GLSZM were present in many models. Gabor filters respond to the edges (changes in the voxel intensities) in an image and give information about the texture in certain directions [14, 48, 49]. GLSZM quantifies gray level zones (i.e., the number of connected voxels that share the same gray level intensity) in an image [14]. The direction of the coefficients in the Elastic Net models indicate that more homogeneous bone texture is associated with osteoarthritis. One explanation for this is that the subjects with osteoarthritis may have thickened subchondral bone which can lead to more homogeneous texture.

One strength of this study was the application of an automatic multi-atlas and appearance model-based algorithm for the segmentation of tibias. Although different MRI sequences and data were used, the performance of our segmentation method was comparable to the previously reported method for the segmentation of tibial bone [50]. It should be mentioned that our method was not fully automatic because 13% of the segmentations needed manual adjustment.

This study has limitations that need to be addressed. First, the radiomic features were extracted only from the tibia. In future studies, features could be extracted from other bones as well. Second, the link between biological processes and individual radiomic features is not always clear. However, MRI-based bone texture has been shown to correlate with the actual three-dimensional microstructure of subchondral bone [19]. Third, only basic risk factors of osteoarthritis were used as covariates in the models. In future studies, models with more risk factors should be used and the additional value of radiomic features on the diagnosis and prognosis of osteoarthritis should be studied.

In conclusion, our results show that radiomic features of tibial bone are different between knees without and with osteoarthritis and could be used as quantitative imaging biomarkers in future studies. As MRI enables assessment of multiple tissues in a joint, extraction of quantitative imaging biomarkers from bone would be beneficial in order to get a comprehensive view of the tissue changes associated with osteoarthritis.

## Supplementary information

ESM 1 (DOCX 1.06 kb)

## Abbreviations

FIESTA: Fast imaging employing steady-state acquisition
GLSZM: Gray level size zone matrix
MOAKS: MRI Osteoarthritis Knee Score

## Declarations

### Guarantor

The scientific guarantor of this publication is Edwin Oei.

### Statistics and biometry

JH and SK have significant statistical expertise related to this study.

### Study subjects or cohorts overlap

The study subjects and the cohort have been previously reported for example in [20–22].

### Methodology

• retrospective

• cross-sectional study

• performed at one institution
