# Supplementary material for: A machine learning approach to distinguish between knees without and with osteoarthritis using MRI-based radiomic features from tibial bone
Source: Eur Radiol. 2021 Apr 21;31(11):8513–21. doi: 10.1007/s00330-021-07951-5 (PMC8523397; doi:10.1007/s00330-021-07951-5)
Supplement: Supplementary file 1 — (DOCX 1.06 kb) [file 330_2021_7951_MOESM1_ESM.docx]

**Supplementary Figure 1**. Receiver operating characteristic and precision-recall curves and respective area under the curve (AUC) values to distinguish between knees without and with osteoarthritis using models with covariates (age and body mass index), image features from medial subchondral bone volume of interest, and covariates and image features in the same model.


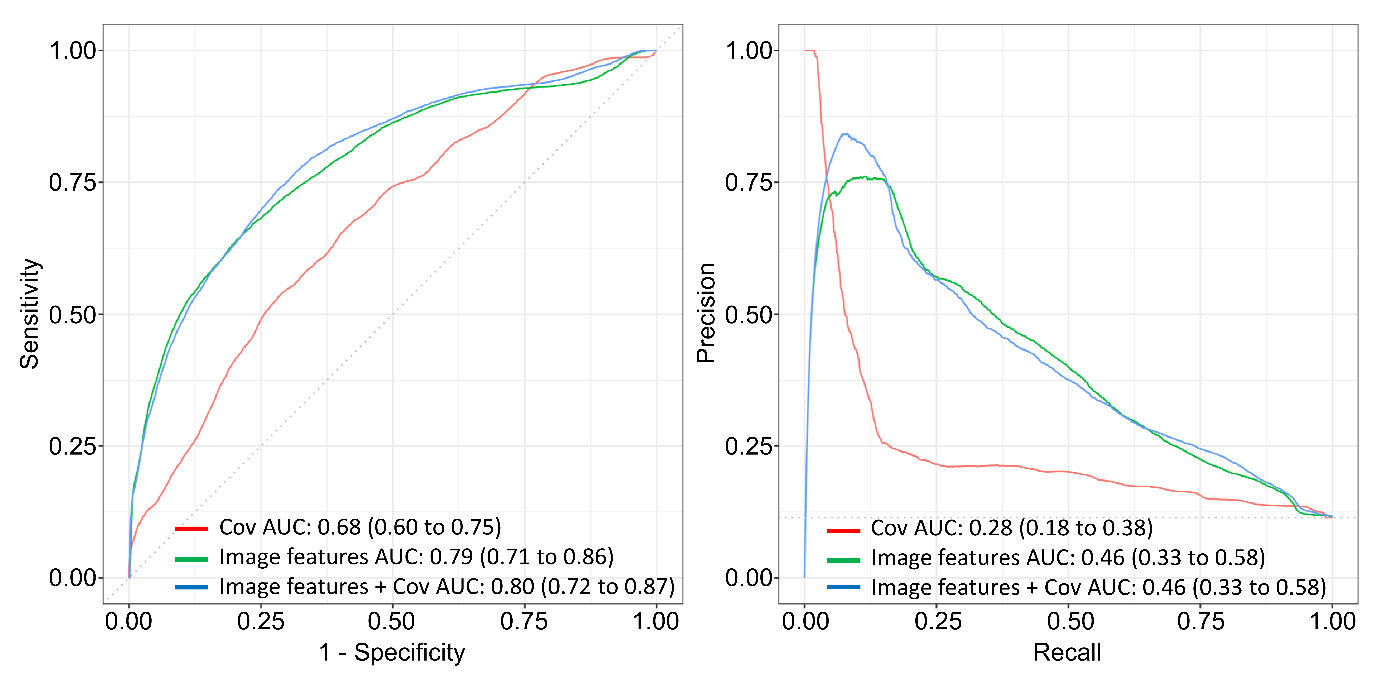


**Supplementary Figure 2**. Receiver operating characteristic and precision-recall curves and respective area under the curve (AUC) values to distinguish between knees without and with medial tibial cartilage damage using models with covariates (age and body mass index), image features from medial subchondral bone volume of interest, and covariates and image features in the same model.


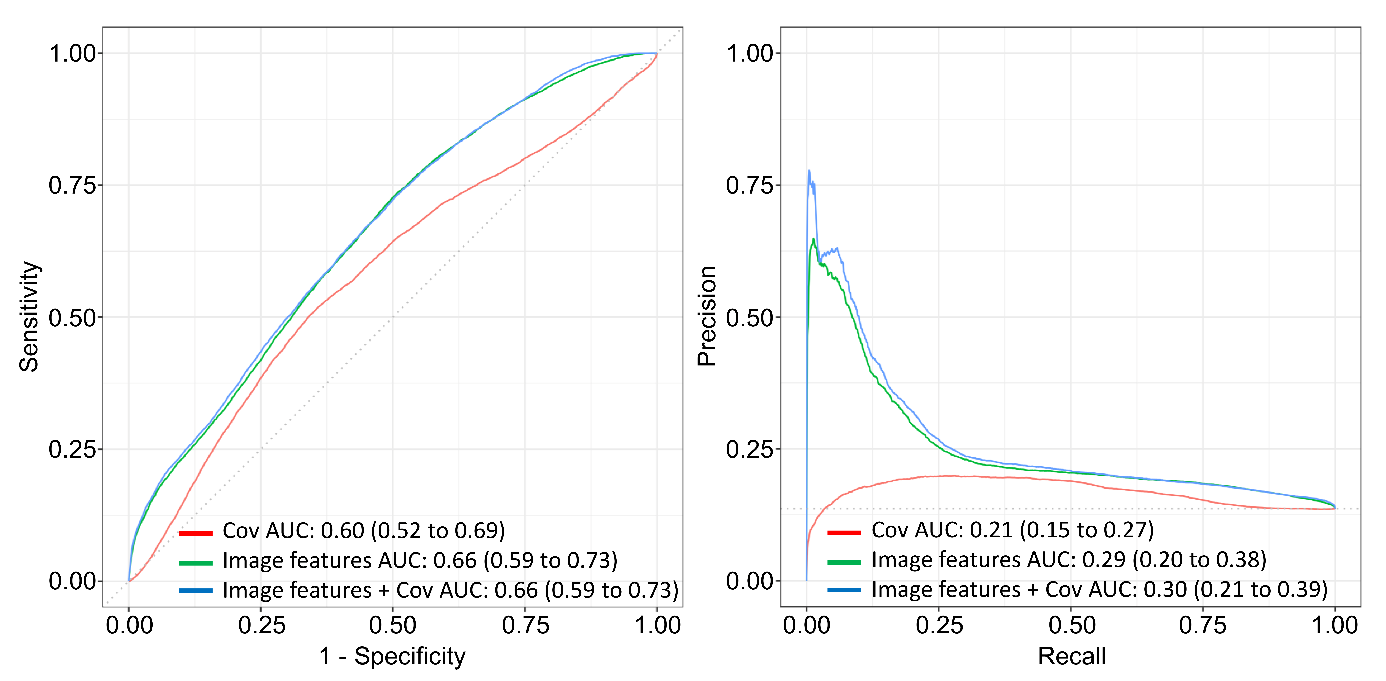


**Supplementary Figure 3**. Receiver operating characteristic and precision-recall curves and respective area under the curve (AUC) values to distinguish between knees without and with medial tibial osteophytes using models with covariates (age and body mass index), image features from all medial side volumes of interests, and covariates and image features in the same model.


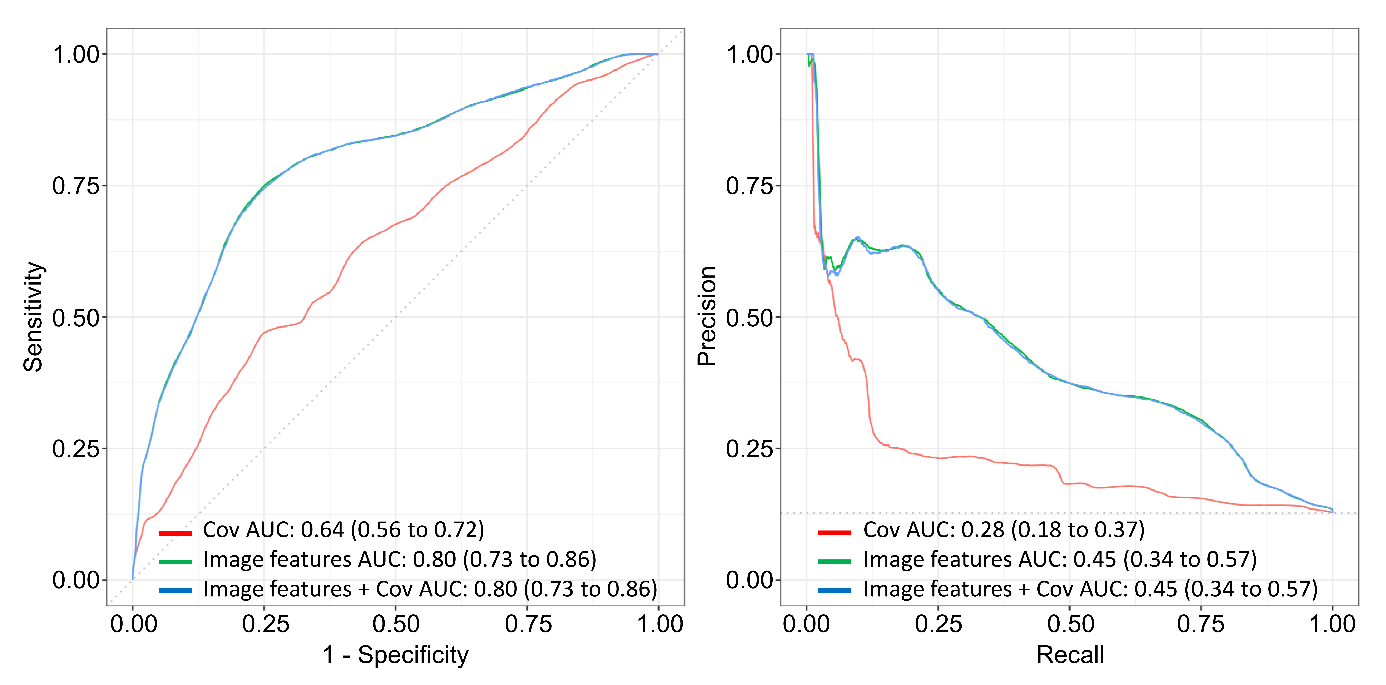


**Supplementary Figure 4**. Receiver operating characteristic and precision-recall curves and respective area under the curve (AUC) values to distinguish between knees without and with medial tibial bone marrow lesions using models with covariates (age and body mass index), image features from medial mid-part volume of interest, and covariates and image features in the same model.


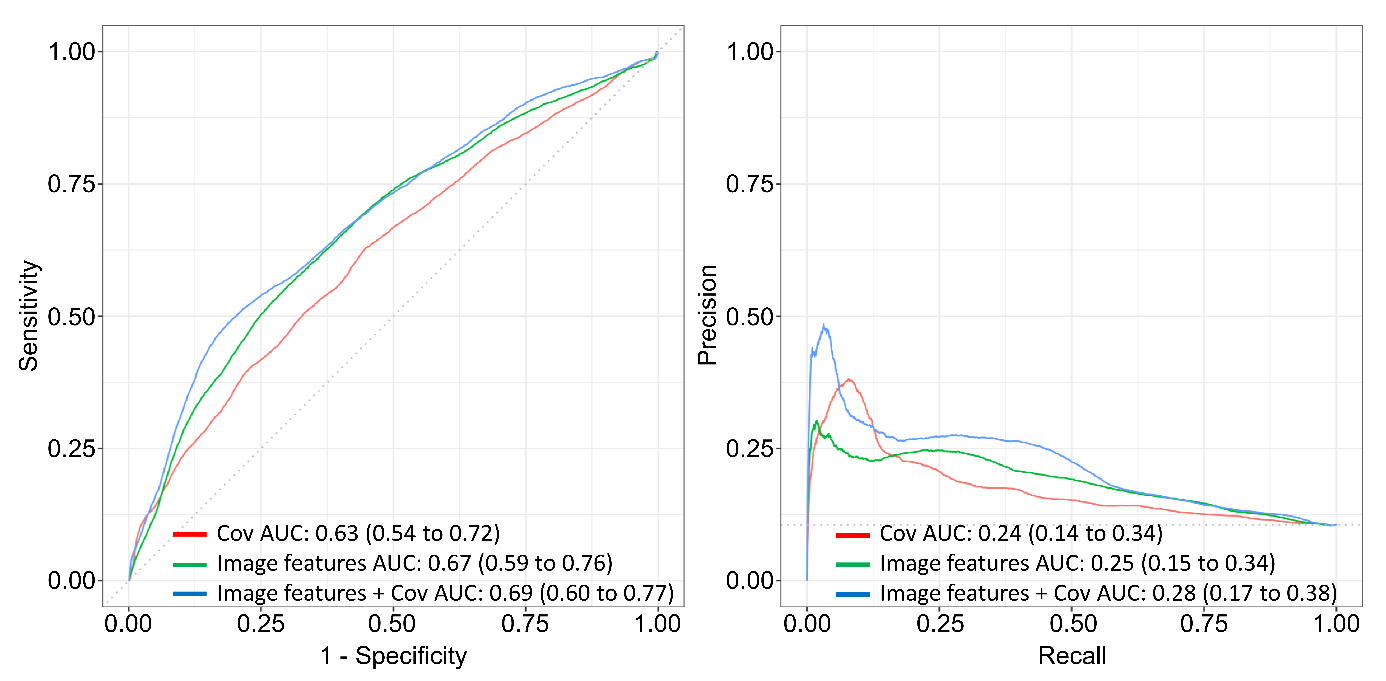


**Supplementary Table 1.** Radiomic features used in the study.

| Shape  (17 features): | Orientation  (3 features): | Histogram  (12 features): | Texture LBP  (6*3=18 features): | Texture GLCM  (6*4*2=48 features): |
| --- | --- | --- | --- | --- |
| compactness (mean + std)  radius (mean + std)  roughness (mean + std)  convexity (mean + std)  circular variance (mean + std)  principal axes ratio (mean + std)  elliptic variance (mean + std)  solidity (mean + std)  volume | theta_x  theta_y  theta_z | min  max  mean  median  std  skewness  kurtosis  peak  range  energy  quartile  entropy | mean  std  median  kurtosis  skewness  peak | contrast  dissimilarity  homogeneity  angular second moment (ASM)  energy  correlation |
| Texture GLCMMS  (12*4*2=96 features): | Texture GLRM  (16 features): | Texture GLSZM  (16 features): | Texture NGTDM  (5 features): | Texture Gabor  (6*4*3=72 features): |
| contrast (mean + std)  dissimilarity (mean + std)  homogeneity (mean + std)  ASM (mean + std)  energy (mean + std)  correlation (mean + std) | GrayLevelNonUniformity  GrayLevelNonUniformityNormalized  GrayLevelVariance  HighGrayLevelRunEmphasis  LongRunEmphasis  LongRunHighGrayLevelEmphasis  LongRunLowGrayLevelEmphasis  LowGrayLevelRunEmphasis  RunEntropy  RunLengthNonUniformity  RunLengthNonUniformityNormalized  RunPercentage  RunVariance  ShortRunEmphasis  ShortRunHighGrayLevelEmphasis  ShortRunLowGrayLevelEmphasis | GrayLevelNonUniformity  GrayLevelNonUniformityNormalized  GrayLevelVariance  HighGrayLevelZoneEmphasis  LargeAreaEmphasis  LargeAreaHighGrayLevelEmphasis  LargeAreaLowGrayLevelEmphasis  LowGrayLevelZoneEmphasis  SizeZoneNonUniformity  SizeZoneNonUniformityNormalized  SmallAreaEmphasis  SmallAreaHighGrayLevelEmphasis  SmallAreaLowGrayLevelEmphasis  ZoneEntropy  ZonePercentage  ZoneVariance | busyness  coarseness  complexity  contrast  strength | mean  std  min  max  skewness  kurtosis |
| GLRLM = gray level run length matrix, GLSZM = gray level size zone matrix, NGTDM = neighborhood gray tone difference matrix, std = standard deviation. Local binary patterns (LBP) features were calculated using the following three parameter combinations: 1 pixel radius and 8 neighbours, 2 pixel radius and 12 neighbours, and 3 pixel radius and 16 neighbours. Gray level co-occurrence matrix (GLCM) and GLCM multislice (GLCMMS) features were calculated in four different directions (0, 45, 90, 135 degrees) using 16 gray levels and pixel distances of 1 and 3. Gabor features were calculated using three different frequencies (0.05, 0.2, 0.5) and four different angles (0, 45, 90, 135 degrees). | | | | |

**Supplementary Table 2.** Hyperparameters (α and λ) and five variables with largest coefficients in the best performing Elastic Net models for classifying knees without and with osteoarthritis, medial tibial cartilage damage, medial tibial osteophytes, and medial tibial bone marrow lesions.

| Model | VOI | α | λ | Variables |
| --- | --- | --- | --- | --- |
| Tibiofemoral osteoarthritis | All | 0.25 | 0.046 | tf_Gabor_0.05A0_minimum in SBM (-), hf_entropy in MidM (+), sf_compactness_mean (-),  age (+), tf_Gabor_0.5A90_kurtosis in SBM (-) |
| Tibiofemoral osteoarthritis | SBM | 0.10 | 0.028 | tf_Gabor_0.05A0_minimum (-), age (+), sf_compactness_mean (-),  tf_Gabor_0.5A0_maximum (-), tf_Gabor_0.5A135_standarddeviation (-) |
| Medial tibial cartilage damage | SBM | 0.65 | 0.010 | tf_Gabor_0.05A0_skewness (+), tf_Gabor_0.05A0_mean (+), tf_Gabor_0.2A45_minimum (+), tf_GLSZM_SmallAreaHighGrayLevelEmphasis (+), tf_Gabor_0.2A0_skewness (+) |
| Medial tibial osteophyte | All | 0.60 | 0.073 | sf_compactness_mean (-), hf_minimum in MidM (-), tf_Gabor_0.05A0_minimum in SBM (-),  tf_GLSZM_GrayLevelNonUniformityNormalized in MidM (-), tf_GLSZM_GrayLevelVariance in MidM (+) |
| Medial tibial osteophyte | MidM | 0.80 | 0.055 | sf_compactness_mean (-), hf_minimum (-), tf_GLSZM_GrayLevelNonUniformityNormalized (-),  tf_GLSZM_GrayLevelVariance (+), tf_GLSZM_HighGrayLevelZoneEmphasis (+) |
| Medial tibial bone marrow lesion | MidM | 0.15 | 0.073 | age (+), hf_kurtosis (-), tf_GLSZM_SmallAreaLowGrayLevelEmphasis (-),  tf_GLSZM_ZoneEntropy (+), of_theta_z (-) |
| VOI = volume of interest, SBM = medial subchondral bone VOI, MidM = medial mid-part VOI, sf = shape feature, hf = histogram feature, tf = texture feature, of = orientation feature, GLSZM = gray level size zone matrix. The numbers in the tf_Gabor variable names indicate the used frequency (0.05, 0.2, 0.5) and angle (0, 45, 90, 135). + and - indicate the sign of the Elastic Net regression coefficient. | | | | |
